# Enhancing Oral Bioavailability of Domperidone Maleate: Formulation, *In vitro* Permeability Evaluation In-caco-2 Cell Monolayers and *In situ* Rat Intestinal Permeability Studies

**DOI:** 10.2174/1567201820666230214091509

**Published:** 2024-03-11

**Authors:** Neslihan Üstündağ Okur, Emre Şefik Çağlar, Mustafa Sinan Kaynak, Mine Diril, Saniye Özcan, Hatice Yeşim Karasulu

**Affiliations:** 1 Department of Pharmaceutical Technology, Faculty of Pharmacy, University of Health Sciences, Istanbul, Turkey;; 2 Department of Pharmaceutical Biotechnology, Faculty of Pharmacy, University of Health Sciences, Istanbul, Turkey;; 3 Department of Pharmaceutical Technology, Faculty of Pharmacy, Anadolu University, Eskişehir, Turkey;; 4 Department of Pharmaceutical Technology, Faculty of Pharmacy, Ege University, Izmir, Turkey;; 5 Department of Analytical Chemistry, Faculty of Pharmacy, Anadolu University, Eskişehir, Turkey

**Keywords:** Domperidone, SEDDS, oral drug delivery, *in situ* intestinal permeability, Caco-2 cell, microbial colonization

## Abstract

**Background:**

The domperidone maleate, a lipophilic agent classified as a Biopharmaceutical Classification System Class II substance with weak water solubility. Self- Emulsifying Drug Delivery System is a novel approach to improve water solubility and, ultimately bioavailability of drugs.

**Objective:**

This study aimed to develop and characterize new domperidone-loaded self-emulsifying drug delivery systems as an alternative formulation and to evaluate the permeability of domperidone-loaded self-emulsifying drug delivery systems by using Caco-2 cells and *via* single-pass intestinal perfusion method.

**Methods:**

Three self-emulsifying drug delivery systems were prepared and characterized in terms of pH, viscosity, droplet size, zeta potential, polydispersity index, conductivity, *etc*. Each formulation underwent 10, 100, 200, and 500 times dilution in intestinal buffer pH 6.8 and stomach buffer pH 1.2, respectively. Female Sprague Dawley rats were employed for *in situ* single-pass intestinal perfusion investigations.

**Results:**

Results of the study revealed that the ideal self-emulsifying drug delivery systems formulation showed narrow droplet size, ideal zeta potential, and no conductivity. Additionally, as compared to the control groups, the optimum formulation had better apparent permeability (12.74 ± 0.02×10-4) from Caco-2 cell monolayer permeability experiments. The study also revealed greater Peff values (2.122 ± 0.892×10-4 cm/s) for the optimal formulation from *in situ* intestinal perfusion analyses in comparison to control groups (Domperidone; 0.802 ± 0.418×10-4 cm/s).

**Conclusion:**

To conclude, prepared formulations can be a promising way of oral administration of Biopharmaceutical Classification System Class II drugs.

## 1 INTRODUCTION

Due to its many benefits, including low risks, high patient compliance, low production costs, a wide range of formulation options, few sterility concerns, and a simple manufacturing process, oral drug delivery has become widespread. However, it also involves limitations in terms of drug delivery related to physicochemical properties of a drug such as poor solubility, low permeability, instability, rapid metabolism, and thus, poor bioavailability, as well as physiological barriers, behaviors, and pH, microbial colonization, and enzymes of the gastrointestinal tract [1-4]. The main determinant of treatment effectiveness is bioavailability, which is influenced by the solubility of drug molecules.

At the same time, solubility is one of the important factors in terms of possessing the desired concentration of the drug in systemic circulation for a pharmacological response. However, according to the pharmaceutical industry, poorly water-soluble drug candidates are becoming more common when the high water-soluble and permeable drug candidates remain at only 8%. The pharmaceutical industry is experiencing an increase in the number of drug candidates with low solubility and high permeability in this context (BCS Class II medicines) [4, 5].

The drug domperidone maleate (DMP), a lipophilic agent classified as a BCS Class II substance with weak water solubility, is rapidly absorbed from the gastrointestinal tract. DMP is a simulation strategy involving cyclodextrins, nanoparticles, solid dispersions, and permeation enhancers to overcome this problem. However, in recent years, it has been observed that using lipid-based formulations improved the oral bioavailability of this type of drug [6, 7]. The most common approach is to incorporate drug molecules into inert lipid vehicles such as oils, surfactant dispersions, self-emulsifying formulations, emulsions, and liposomes. It has piqued the interest of pharmaceutical researchers [8].

Self- Emulsifying Drug Delivery System (SEDDS) is a novel approach to improving water solubility and, ultimately bioavailability of drugs [9]. SEDDS are isotropic mixtures of drugs, lipids, and surfactants, usually with one or more hydrophilic co-solvents or co-emulsifiers [8]. These systems can instantaneously form fine oil-in-water emulsion upon mild agitation followed by dilution with aqueous media. The droplet size of these systems can range from nanometers to microns [10]. Since DMP is a BCS Class II medicine, this study aimed to enhance its bioavailability and patient compliance by lowering the drug's release time from the formulation. Therefore, DMP-loaded SEDDSs were prepared, characterized, and tested for permeability utilizing *in vitro* Caco-2 cell monolayer and *in situ* single-pass intestinal perfusion method.

## 2 MATERIALS AND METHODS

### 2.1 Materials

Deva Holding (Turkey) donated DMP. From Merck-Germany, we bought oleic acid, Phenol Red (P.R.), PEG 400, Span 80, and Tweens 20 and 80. Gattefosse-France provided Labrasol, Capyrol-90, Labrafac-PG, and Transcutol-HP. Novartis donated metoprolol tartrate (MTT) (Turkey). Cremophor EL, acetonitrile, methanol, ortho-phosphoric acid, and the materials for golytely solution (sodium chloride, potassium chloride, sodium sulfate, sodium hydroxide, and mannitol) were supplied by Sigma-Germany.

### 2.2 Methods

#### 2.2.1 Screening of Excipients

To detect optimum formulation components of SEDDSs, the solubility of DMP was determined in distilled water, ethanol, Span 80, PEG 400, Tween 20, Tween 80, Cremophor EL, and oleic acid, Labrasol, Transcutol, Capyrol 90, Labrafac PG, 0.1M HCL, gastric and intestinal medium at 25 ± 2°C. An excess amount of the drug was added into 2 mL excipients and mixed for 72 h at 25 ± 2°C. HPLC was used for the determination of drug concentration.

#### 2.2.2 Determination of N-octanol/Water Partition Coefficient

The saturated n-octanol-water mixture was supplemented with 10^-3^M DMP to calculate the DMP's n-octanol/water partition coefficient. After being stirred for 24 hours, the samples were centrifuged at 4000 rpm. To measure the drug concentration in the water phase, HPLC was used.

#### 2.2.3 HPLC Analysis

For the characterization, *in vitro* dissolution, and cell culture studies, the HPLC system consisted of a U.V. detector supplied by Agilent 1100 (USA). C18 column (5 µm, 150x4.6 mm) was used. For the characterization and *in vitro* dissolution studies, the samples were analyzed at 270 nm with 1 mL/min at a 25 ± 2°C flow rate. The injection volume of the samples was 20 µL. The mobile phase was a mixture of acetonitrile and methanol (30:70). The samples were analyzed at 230 nm with 2 mL/min at a 27 ± 2°C flow rate for cell culture. Phosphate buffer (50 mM, pH 3.5) and acetonitrile (80:20, v/v) were utilized as the mobile phase [11].

Shimadzu's Prominence series of HPLC system was used to analyze *in situ* single-pass intestinal perfusion studies. The stationary phase was a G.L. Sciences Intersil ODS-3 column (150×1.5 mm, 5.0 μm). The samples were analyzed at 206 nm with a 1.2 mL/min flow rate. A mixture of phosphate buffer: methanol (43:57) in isocratic mode comprised the mobile phase

#### 2.2.4 Preparation of DMP-loaded SEDDS

Oleic acid was used as the oil phase, Labrasol, PEG 400, Tween 80 as a surfactant, and Transcutol HP as the co-surfactant to prepare the SEDDS. For each phase diagram for a given weight ratio of surfactant and cosurfactant, the ratio of oil to the mixture was varied from 1:1 (w/w), 1:2 (w/w), 1:3, 1:4, 2:1 (w/w), 3:1 (w/w), and 4:1 (w/w). At 25°C, these mixtures were titrated with distilled water drop by drop as they were gently swirled. Selecting formulations at the desired component ratios required first locating the SEDDS region in the phase diagrams. Each 5 g SEDDS was loaded with 10 mg of DMP.

#### 2.2.5 Characterization of SEDDS

Malvern Zetasizer was used to measure the droplet size and polydispersity index (PDI) using the dynamic light scattering method (Nano ZS, Malvern Instruments, U.K.). 10 measurements at an angle of 173° at 25°C were averaged to get the droplet size and PDI values [12], using disposable cells.

The formulations' pH was measured using a pH meter (Mettler Toledo, Switzerland). Determinations were carried out in triplicate. A viscosimeter (AND Viscometer-SV-10, USA) measured the viscosities of formulations. Experiments were performed five times for each sample. The electrical conductivity of the microemulsions was studied at 25 ± 2°C using a conductometer (Hanna Ins., USA). Experiments were performed in triplicate [12].

#### 2.2.6 Robustness to Dilution

The robustness of formulations was examined by diluting 1:10, 1:100, 1:200, and 1:500 ratios with gastric buffer pH 1.2 and intestinal buffer pH 6.8. Any evidence of phase separation or precipitation in the diluted samples was noted. Additionally, the droplet size of the formulation in each sample was measured [13, 14].

#### 2.2.7 Stability

For physical stability, formulations were tested by centrifugation. The formulations were centrifugated at 5.175×g for 30 minutes and observed for any phase separation. Studies on pH measurement, phase separation, and clarity were also carried out. The concentration of DMP in the formulations was also assessed during the three-month chemical stability tests using HPLC analysis.

5 g of each formulation (S1-DMP, S2-DMP, and S3-DMP) were placed in amber-colored glass vials for tests, including heating and cooling cycles. Six cycles of 40°C for 24 hours in the incubator and 5°C for 24 hours in the refrigerator were applied to the samples. These formulations were centrifuged at 3,000 rpm for five minutes. Research into turbidity and phase separation was conducted.

#### 2.2.8 *In vitro* Dissolution Studies

*In vitro* dissolution studies were performed according to dissolution apparatus (Pharma Test PTWS 820D, Germany) II in USP 24. 900 mL of simulated intestinal medium (pH 6.8) was added to dissolution vessels, which were then heated to 37°C and agitated at 50 rpm. The dissolving medium was sampled (1 mL) at predefined intervals. The commercial tablet formulation of DMP containing the same amount of drug and the SEDDS formulation's DMP dissolution were compared. At 270nm, HPLC was used to examine the samples.

#### 2.2.9 Permeability Studies in Caco-2 Cell Monolayer

##### 2.2.9.1 Caco-2 Cell Culture

For the permeability investigations, Caco-2 cells were received from the American Type Culture Collection (ATCC). The Dulbecco's Modified Eagle medium (DMEM) cultivated the cells. The medium contained 450 mL of L-glutamine (1%, v/v, 2 mM), 50 mL of fetal bovine serum, 5 mL of Hank's Balanced Salt Solution (HBSS), 5 mL of alanine, and 5 mL of penicillin (100 IU/mL). From passage 21 to passage 55, Caco-2 cells were grown. Cell monolayers were created by seeding 4x105 cells per 4.67 cm2 on six transwell insert filters for permeability. 37°C cell culture kept in a humid environment with 90% humidity and 5% CO_2_. 20-22 days after sowing, monolayers were used. Each cell monolayer's integrity was examined by measuring its transepithelial electrical resistance (TEER) before and after the experiment using an epithelial voltameter (EVOM, World Precision Instrument, Sarasota, FL). The Eq. (1) below was used to calculate the TEER value:


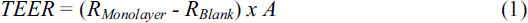


Where RMonolayer denotes the resistance of the filter membrane alone, RBlank denotes the resistance of the filter membrane, and A denotes the membrane's surface area (4.67 cm^2^ in six-well plates).

##### 2.2.9.2 Permeability Studies

**Fig. (1):**
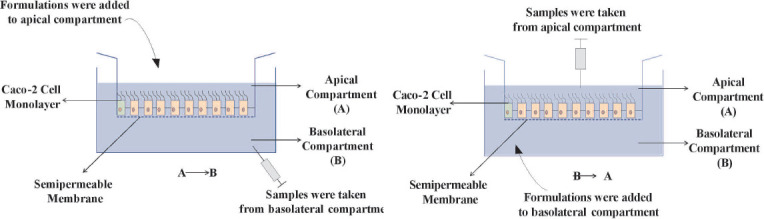
Schematic presentation of the permeability study through Caco-2 cell monolayer.

The *in vitro* permeability analysis used Caco-2 cell monolayers grown in transwell inserts with collagen-coated polycarbonate membranes having a pore size of 0.4 µm and a surface area of 4.67 cm^2^. The cells were kept in the environment at 37°C. For three weeks, the medium was changed every other day. DMP-loaded SEDDS formulations, DMP solution, and suspension of commercial tablet in HBSS were employed for the Caco-2 cell monolayer tests (Fig. **1**).

Studies on permeability were conducted from apical to basolateral (A→B) and from basolateral to apical (B→A) in both directions. Formulations, DMP solution, and suspension of commercial tablets were administered to the apical side (A, 2 mL) for (A→B) direction or to the basolateral side (B, 3 mL) for (B→A) direction after twice washing the Caco-2 cell monolayer with HBSS medium (pH 7.4). For the transport studies, the six-well plates containing the cell monolayer were put into an orbital environmental shaker maintained at a constant temperature (37°C) and an agitation rate of 50 rpm (2 h). At 30-minute intervals, four sequential samples of 200 L each were drawn from the acceptor compartment and subjected to HPLC analysis.

Apparent permeability values (P_app_) for each side were calculated according to the following Eq. (2):


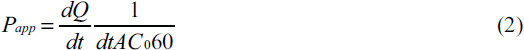


Where the P_app_ is the apparent permeability (cm/s), dQ/dt is the permeability rate, A is the diffusion area of monolayers (cm^2^), and C_0_ is the initial concentration of the drug in the donor compartment [1, 2].

#### 2.2.10 Perfusion Solution for SPIP Studies

For SPIP tests, perfusion solution (Golytely solution) was made using 5 mM NaCl, 10 mM KCl, 40 mM Na_2_SO_4_, 20 mM NaHCO_3_, and 80 mM mannitol. The buffer's pH was adjusted to 7.4 with orthophosphoric acid or a KOH solution. A 0.45 µm membrane filter was used to filter the newly prepared perfusion medium [3]. Purified water (Milli-Q) was added to each component to dissolve it completely. The concentration of each drug or marker (Table **1**) used in the perfusion experiments was determined by dividing the maximum recommended dose by 250 mL. This volume should be consumed to represent the highest medication concentration in the gut accurately [3-5].

#### 2.2.11 *In situ* SPIP Assays and Surgical Procedure

The Committee for the Use and Care of Animals at Anadolu University authorized all protocols used to conduct animal studies (Protocol No. 2020/41). All *in situ* intestinal perfusion investigations employed female Sprague Dawley rats weighing 200-250 g. The rats were given free access to tap water and fasted for around 12 hours the night before each trial.

According to previously reported investigations and published publications, the experimental process for the *in situ* SPIP studies was carried out [3, 6]. An intraperitoneal injection of a ketamine-xylazine mixture (90-10 mg/kg, respectively) was used to anesthetize the rats. Every animal is put on a heated surface that is kept at 37 ± 0.5°C. A midline (3-4 cm) incision was made in the belly to expose the small intestine. The flexible PVC tubing (inlet tubing, internal diameter (id) 0.76 mm; outlet tubing, id 1.70 mm) was carefully cannulated into the jejunal segment (10 cm), and the tubings were then connected to the perfusion system. Care was taken, and exposed segments were kept wet with 37°C saline solution to retain an intact blood supply to the segment. Perfusion medium was incubated at 37 ± 0.5°C in a water bath. The surgical site was then wrapped with parafilm to prevent fluid from exposed segment surfaces from evaporating. The peristaltic pump (Minipuls-3, Gilson, France) was used to pump the blank perfusion solution through the gut at a flow rate of 0.5 mL/min for about 20 min. To clear out any remaining debris. The cannulated intestinal segment was cleaned before the perfusion solution containing the drugs/markers was administered for 60 minutes at a flow rate of 0.2 mL/min. In tubes, samples were taken from the distal region of the jejunum at intervals of 10, 20, 30, 40, 50, and 60 minutes.

After collection, samples were immediately frozen at -20°C for high-performance liquid chromatography analysis (HPLC). According to standards for euthanasia in experimental animals, animals were cervical dislocated to end each experiment. Following each experiment, the intestinal segment's length was measured.

#### 2.2.12 Determination of Effective Intestinal Permeability (P_eff_) Fraction Absorbed in Humans of DOMP

For calculating the drug's effective permeability values (Peff), the measured C_out_/C_in_ ratio was corrected for water transport using Eq. (3) [7].


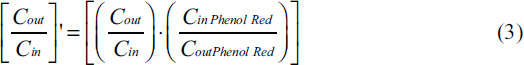


where C_in Phenol Red_ is the inlet P.R. concentration, and C_out Phenol Red_ is the outlet P.R. concentration.

The effective permeability (P_eff_) values of the medication across the rat gut wall were computed [14] using the “plug flow” model as shown in Eq. (4).


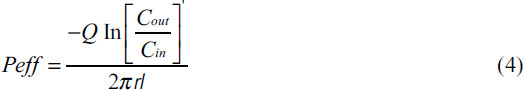



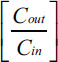
 is the adjusted drug concentration ratio of outflow to inlet concentration, and Q is the flow rate of the perfusion solution (mL/sec) (Eq. 4). For the jejunum, the radius of the perfused intestinal segment is r = 0.2 cm, and the length is l (cm) [4, 8, 9].

Net water flux (NWF) values were calculated based on inlet (C_in Phenol Red_), outlet (C_out Phenol Red_) concentrations of P.R. and (Q_in_) the inlet perfusate flux using the following Eq. (5):


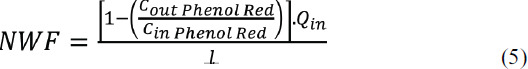


A negative net water flux indicates fluid loss from the mucosal side (lumen) to the serosal side (blood). A positive net water flux indicates fluid secretion into the segment [10].

The effective permeability values for humans (P_eff humans_) were calculated using P_eff rats_ values obtained from *in situ* rat SPIP studies, according to Eq. (6) proposed by Fagerholm *et al*. [11].


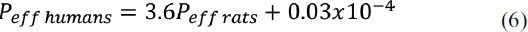


where _eff humans_ = effective permeability predicted for humans, P_eff rats_ = effective permeability obtained in rats using the SPIP method. The fraction absorbed (F_a_) in humans can be calculated for each substance using Eq. (7), presented by Sugano *et al*.


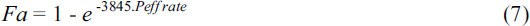


Where F_a_ = fraction absorbed predicted for humans P_eff rats_ = effective permeability obtained in rats by the SPIP technique.

### 2.3 Statistical Analysis

The means and standard deviations of all outcomes were reported (S.D.). The Kruskal-Wallis test was employed for multiple comparisons. When necessary, the two-tailed non-parametric Mann-Whitney U test was employed to compare the two experimental groups and find any differences. A p-value of less than 0.05 was regarded as significant.

## 3 RESULTS AND DISCUSSION

### 3.1 Screening and Octanol/Water Partition Coefficient Studies

For the formulations of SEDDSs, screening oil, surfactant, and cosurfactant is necessary to obtain an optimum preparation. It is important to find convenient components to dissolve the DMP for enhanced drug solubility to obtain optimum SEDDS formulations. Moreover, with the maximum solubilizing potential of the drug in an oil and surfactant mixture, the efficacy of drug loading can be increased, and the final volume of SEDDS can be minimized [12].

Fig. (**2**) displays the drug's solubility in several common oils, surfactants, and co-surfactants. It was found that DMP was most soluble in PEG 400 (8.622 ± 0.009 mg/mL), followed by Tween 20 (5.898 ± 0.009 mg/mL). For this study, nonionic surfactants PEG 400, Tween 80, and Labrasol were chosen due to advantageous features such as high solubility, less toxicity, less hemolytic and irritating effects, and high stability [13]. Due to its high hydrophilic-lipophilic balance value (HLB), Tween 80 (HLB 15) is one of the most widely used nonionic surfactants [14].

The surfactants with high HLB values (especially HLB 12-15) are preferred to be chosen because they have good efficiency of self-emulsification. Indeed, self-emulsification is much related to the HLB values of the surfactant [15]. Labrasol, a medium-length alkyl-chained surfactant, was reported for its improved intestinal absorption of drugs [16]. PEG 400 and Transcutol HP showed the drug's highest solubility and improved ability to mix with other components.

It has been demonstrated that a drug's aqueous solubility and lipophilicity affect its pharmacokinetic profiles, therapeutic activity, and membrane flux. The log P of n-octanol/water can be calculated to determine the drug's lipophilicity. The Log P value of the DMP was determined as 1.2880 ± 0.0314. This value indicates that DMP has moderate lipophilicity.

### 3.2 Preparation and Characterization of DMP-loaded SEDDS

A pseudo ternary phase diagram was constructed to identify the SEEDS area by assessing the composition's self-emulsifying abilities and the likely concentration of oil, surfactant, and co-surfactant required to create a stable SEEDS. The pseudo-ternary phase diagrams for drug-free SEDDS formulations are shown in Fig. (**3**).

The concentration of the components (oil, surfactant/co-surfactant, and water) was shown in the pseudo-ternary phase diagrams as a weight-to-weight percentage (w/w%). The region for self-emulsification was demonstrated as the shadow areas in the phase diagram. Many studies showed that the pseudo-ternary phase diagrams were drawn using the water titrating method [15, 17, 18]. Formulations in this study were also developed by using a water titrating methodology.

The regions in the diagrams' sizes were compared; the larger size, the higher the effectiveness of self-emulsifi-cation. The self-emulsification attitude was more pronounced in Formulations S1 and S3.

This study used safe components involving oleic acid as the oil phase, Tween 80, Labrasol, PEG 400 as surfactant and Transcutol as co-surfactant. The concentration of each ingredient used in the manufacture of SEDDSs is shown in Table **2**. For formulations S1, S2, and S3, the surfactant/co-surfactant ratio (S1: Tween 80/Transcutol, S2: Labrasol/Transcutol, S3: PEG 400/Transcutol) was found 1:1, 3:1, 1:2 respectively.

After identifying the self-emulsification region, DMP was slowly dissolved in the system under stirring, and clear SEDDSs were obtained. The concentration of DMP for each formulation was 2 mg/g.

As shown in Table **2**, all three formulations have the same oil phase and cosurfactant; however, the surfactant varies depending on the formulation. Tween 80, Labrasol, and PEG 400 are the surfactants used in the S1 formulation, S2 formulation, and S3 formulation. All three of the surfactants are non-ionic and are used in formulations. Since non-ionic surfactants are less affected by changes in pH and ionic strength, they are extremely beneficial for formulation design. Additionally, reducing the droplet size of the formulation by adding a non-ionic surfactant is crucial for the drug's bioavailability [19, 20].

Furthermore, non-ionic surfactants, such as Tween 80, Labrasol, and PEG 400, inhibit p-glycoprotein activity, increasing bioavailability. This is why choosing a non-ionic surfactant for oral self-emulsifying drug delivery is such an important factor to consider [21-25].

The cosurfactant makes the interfacial film more fluid, which helps form an emulsion. Transcutol HP is thought to be more effective and can easily encourage water penetration at interfaces due to its shorter chain length (C6) [26]. In addition, to facilitate drug diffusion of the submicron-sized droplets through the mucosal barrier on the one hand, as well as serving as a solubilizer for DMP, a well-known permeation enhancer as Transcutol HP was added to the formulations [27].

Viscosity, conductivity, and pH studies were performed for blank and DMP-loaded formulations. Viscosity values of the formulations (S1, S1-DMP, S2, S2-DMP, S3, S3-DMP) were found 60.44 ± 0.049, 56.08 ± 0.04, 50.68 ± 0.04, 48.94 ± 0.102, 20.06 ± 0.215, 19.9 cP, respectively. All formulations showed no conductivity since all formulations contain non-ionic surfactants. In addition, pH values of S1, S1-DMP, S2, S2-DMP, S3, S3-DMP formulations were measured as 7.5 ± 0.058, 7.5, 6.3 ± 0.063, 6.46 ± 0.049, 8.32 ± 0.04, 7.22 ± 0.075, respectively.

### 3.3 Robustness to Dilution

Uniformity of SEDDS at different dilution ratios is very important in drug absorption. It is because drugs can precipitate at higher dilutions *in vivo*, and precipitation of the drug significantly changes the absorption rate and ratio [28]. Different fold dilutions of selected formulations were exposed to different media to mimic the *in vivo* conditions where the formulation would encounter gradual dilution. Hence, each formulation was subjected to 10, 100, 200, and 500 times dilution in gastric buffer pH 1.2 and intestinal buffer pH 6.8.

Results (Table **3**) showed that the droplet size of formulations increased with the dilution ratio but did not significantly change. Formulations were diluted and kept for 4 and 12 hours. The stability of the reconstituted formulations was guaranteed by the absence of phase separation, cloudiness, and drug precipitation even after 12 hours had passed. Every formulation displayed comparable droplet sizes in all dilutions and mediums. Additionally, it was discovered that all formulations' PDI values were lower than 0.5.

### 3.4 Stability Studies

Thermodynamic stability studies were performed to evaluate phase separation and the effect of temperature variation on SEDDS formulations. Formulations were observed visually for phase separation [29]. After a 30-minute centrifuge test on the S1-DMP, S2-DMP, and S3-DMP formulations, no symptoms of phase separation were seen. However, after 3-month the S2-DMP formulation showed phase separation. Clarity and pH changes were not observed for 3 months. The concentration of DMP in S1 and S3 formulations remained constant, and no degradation was observed during storage.

A test that involved heating and cooling the formulation was conducted. S1-DMP, S2-DMP, and S3-DMP all passed the heating and cooling cycle test. Not all formulations showed symptoms of phase separation.

### 3.5 *In vitro* Dissolution Studies

According to Dissolution Apparatus II in USP 24, the *in vitro* dissolution investigation was carried out. The disintegration profiles of each SEMDDS and commercial tablet (Motilium ^®^) are shown in Fig. (**4**).

The release pattern of SEDDS reveals that the maximum drug release was observed with formulation S2-DMP after half an hour, but at the end of the 4th hour, it was found that formulation S2-DMP went for phase separation. This is the rationale behind the permeability tests that were carried out utilizing the formulations S1-DMP, S3-DMP, conventional tablets, and DMP solution on Caco-2 cell monolayers. On the other hand, once formulation S1 showed 100 ± 4.176% release, formulation S3-DMP showed 80.75 ± 3.250% of the drug was released. Furthermore, 52.58 ± 2.866% of the drug was released from the conventional tablet at the end of the third hour. These results vary due to the composition of oil and surfactant in the system. The formulation S3-DMP showed less drug release when compared to the other SEDDS formulations; this may be due to a higher proportion of oil resulting in larger droplet size, leading to lesser surface area exposed to the medium [30]. The data indicate that the release rate of domperidone maleate from SEDDS formulations was considerably faster than the marketed drug formulation. The cumulative percentage drug release from the S1-DMP and S3-DMP formulations was found to be 100.00 ± 4.18% and 80.75 ± 3.25%, respectively; this is in contrast to the marketed formulation, which had a release of 52.58 ± 2.87% over 4 hours. Thus, it can be concluded from *in vitro* data that the produced SEDDS formulations demonstrated enhanced DMP solubility. These findings also demonstrated that when DMP is loaded into SEDDS, and the oil droplets are absorbed by various oil absorption methods such as passive diffusion, pinocytosis, or endocytosis, it is possible to achieve greater DMP absorption in the gastrointestinal system. Furthermore, the small droplet sizes of the formulations expose a large amount of interfacial surface area for drug release and absorption, resulting in higher bioavailability [31-35].

### 3.6 Domperidone Maleate Permeability Studies in Caco-2 Cell Monolayer

#### 3.6.1 Permeability of Domperidone Maleate

Oral drug delivery is always easy to deliver to the human body. Due to this, the oral administration of the drugs has the highest patient compliance. In this regard, understanding oral absorption of the drug in humans is important in developing new formulations for oral administration. The intestinal permeability values are calculated after the oral administration of the drug to understand the drug absorption in the small intestine. The Caco-2 cell permeability test is a suitable method for evaluating the transport efficacy of novel formulations in such research [33, 36, 37]. Differentiated Caco-2 cell monolayers can develop effective tight junctions and have similar morphological and biochemical features to the intestinal enterocytes. Due to it, the cell line is very popular among pharmaceutical technologists as an *in vitro* model of the intestinal mucosa to determine the transport characteristics of the formulations and, therefore, to design formulations with enhanced membrane permeability [38].

This study assessed the permeability of created SEDDSs formulations (S1 and S3), DMP solution, and commercially available product across Caco-2 cell monolayers. The values of the permeability coefficient (Papp) for the commercial tablet and solution formulations of S1-DMP and S3-DMP are shown in Table **4**. Additionally, Fig. (**5**) displays the concentration of DMP that has permeated through the Caco-2 cell monolayer from SEDDS, solution, and commercial product.

Mostly, substances with apparent permeability coefficients less than 1x10-6 cm/s are considered low permeability substances. For substances that show medium permeability, Papp values are between 1x10^-6^ and 1x10^-5^ cm/s, and high permeability substances exhibit apparent permeability coefficients of > 1x 10^-5^ cm/s [36].

According to the study, commercial tablet and solution permeability values for DMP were lower than those obtained from SEDDS formulations. For all formulations, the permeability from the basolateral to apical direction was greater than the apical to basolateral permeability values. Papp (A→B) value for each formulation was found above 1x 10^-5^ cm/s. DMP was thus expressed in the formulations to have a high permeability.

The efflux ratios of DMP [Papp(B→A)/Papp(A→B)] for all formulations are shown in Table **4**. When the efflux ratio of the drug material is less than 2, no transporter is involved in the permeability process of drug compounds across Caco-2 cell monolayers [39]. Prepared formulations in this investigation showed efflux ratios lower than 2. This suggests a passive transport mechanism for the SEDDS formulations employed in this study.

#### 3.6.2 Transepithelial Electrical Resistance (TEER) Measurements

Conducting cell viability studies is essential for permeability studies since the substances composing SEDDS formulations act as absorption enhancers and alter the epithelial barrier properties. TEER measurements were used following transport tests to examine the impact of formulations on the Caco-2 cell monolayer integrity. Before and after the experiments, the cells' TEER resistance was measured as shown in Fig. (**6**).

Before the trial, the TEER values of the formulations ranged from 1002.778 ± 8.075 to 1107.222 ± 35.766 (ohms/cm). Following the experiment, TEER tests revealed TEER values ranging from 611.367 ± 28.453 to 1107.867 ± 52.313 (ohms/cm). For the A→ B direction, the percentages of TEER change for S1 and S3 formulations were 1.5% and 7.9%, respectively. For the B→ A direction, the percentage of TEER change for both S1 and S3 formulations was 12.3%. The changes in TEER values were greater for B→ A direction when compared to the A→ B direction. The effect of DMP-loaded formulations, solutions, and commercial tablets are presented in Table **5**. Despite TEER changes being observed, the cell monolayer was not damaged because the range of TEER values did not exceed 40%. After completing permeability tests, this demonstrated the viability of Caco-2 cells [40].

#### 3.6.3 *In situ* Intestinal Rat Permeability Studies of Domperidone Maleate

The cannulation procedures of the intestinal segment were carefully performed to maintain the intestinal segment's physiological blood flow and nerve ligaments' physiological positions and to ensure normal physiological conditions. The perfusion flow was set to 0.2 mL/min.

The intestinal membrane integrity was measured using P.R., a zero-permeability marker. This marker is widely used in different models of permeability studies [41].

Net water flux (NWF) values indicated the loss of fluid from the mucosal side (lumen) to the serosal side (blood) in the pure DMP group (-47 ± 35 mL/h/cm), DMP tablet group (-35 ± 13 mL/h/cm) and DMP Loaded S3 group (-30 ± 14 mL/h/cm). Water secretion of fluid into the segment was observed for the Blank S3+DMP group (23 ± 18 mL/h/cm) (Table **6**).

Since the absorbent clearance values for chemicals determined from *In situ* Perfusion tests depend on loss from the intestinal lumen, Yang *et al*. believe that NWF must be utilized to modify the effluent concentration. Although NWF may play a role in determining absorptive clearance, for drugs with low permeability, the clearance can be approximated using a non-linear equation that approaches a linear connection even if NWF does play a role [42].

#### 3.6.4 Permeability of Domperidone Maleate

In all the groups, MTT was administered as a marker to ensure the experiment was performed properly, and P.R. was utilized as a non-absorbable marker. It is simultaneously perfused with MTT to assess DMP permeability. According to the findings (Table **7**), permeability (P_eff_) was 0.376 ± 0.092x10^-4^ cm/s for MTT and 0.802 ± 0.418x10^-4^ cm/s for DMP.

The S3-DMP had the highest P_eff_ and k_a_ values, whereas tablet and pure drug were the least permeable forms (Table **7**).

The increment in P_eff_ and k_a_ was expected with DMP-loaded SEEDSs. In one study, docetaxel-loaded SEDDS formulation was prepared and evaluated for intestinal permeation. According to the results, it was found that effective permeability was significantly higher when compared to the control group [43]. In another study, low soluble drug piperine was loaded with SEDDS formulations. Results showed that the piperine-loaded SEDDS significantly increased drug permeability compared to control groups [44]. To a large extent, our data corroborated these findings. P_eff_ of DMP was enhanced by roughly 265% in our study, as expected.

Drugs administered in nano-lipid carriers may have increased permeability and bioavailability for a variety of reasons, including (i) maintaining the drugs' solubilized state in the G.I. tract; (ii) forming mixed micelles; and (iii) encouraging the secretion of endogenous phospholipids and bile salts [45].

**Fig. (7):**
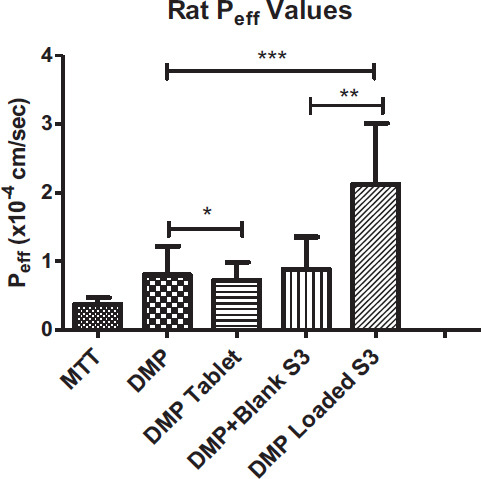
The permeability co-efficient (Peff, cm/sec) of DMP obtained from perfusion of rat ileum (mean S.D., n=6). *p>0.05, DMP *vs.* DMP tablet, **p<0.05, DMP+Blank S3 *vs.* DMP Loaded S3, ***p<0.05, DMP *vs.* DMP+Blank S3 and DMP Loaded S3.

**Fig. (8):**
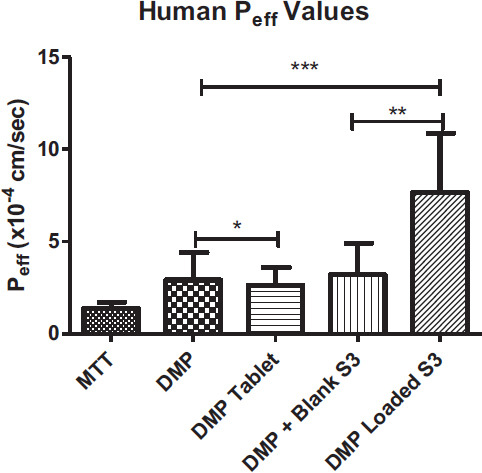
Calculated human P_eff_ values *p>0.05, DMP *vs.* DMP tablet; **p<0.05, DMP+Blank S3 *vs.* DMP Loaded S3; ***p<0.05, DMP *vs.* DMP+Blank S3 and DMP Loaded S3.

These mechanisms might be used to explain the enhanced absorption obtained by DMP-loaded S3. However, it was discovered that the Peff value of DMP+Blank S3 was practically identical to the Peff values of pure DMP and DMP-Tablet. As a P-glycoprotein (P-gp) substrate, DMP administration without formulation limits DMP absorption since P-gp exists [46]. In one study, researchers looked at the impact of piperine, a P-gp inhibitor, on the absorption of DMP. The findings revealed that the DMP absorption was considerably higher in the group that had received piperine pretreatment. According to findings from a different study, the SEDDSs composition can influence P-gp function and even operate as a P-gp inhibitor. Because of this, the S3 formulation's composition may impact P-gp activity in DMP absorption [46] (Figs. **7** and **8**).

In addition, as seen in Fig. (**9**), fractional absorption of DMP-loaded S3 formulation was significantly increased compared to control groups.

## CONCLUSION

In this study, DMP-loaded SEDDS were successfully prepared by constructing a pseudo-ternary phase diagram and optimized with an easy technique. The DMP SEDDS achieved good stability, narrow size distribution, and low PDI. In addition, an *in vitro* dissolution study was conducted to evaluate the dissolution profiles of DMP, a poor solubility BCS Class II drug. The results showed a significant enhancement of *in vitro* dissolution for the drug-loaded SEDDS compared to the commercial tablet. Permeability results obtained from Caco-2 cell monolayer studies showed that the S3-DMP showed almost 2.4 times higher permeability when compared to the marketed product. In addition, SPIP studies revealed that the permeability of DMP from the S3 formulation was 3.44 times higher when compared to the marketed product. The developed SEDDSs, and the S3-DMP formulation, show promise for the oral delivery of BCS class II drugs for this reason. Hence, it can be concluded that SEDDS can improve the solubility and intestinal absorption of DMP, a poorly water-soluble drug.

## Figures and Tables

**Fig. (2):**
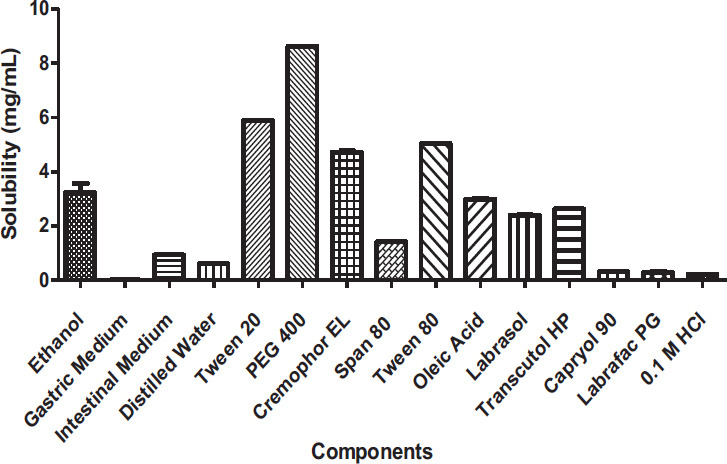
The solubility of domperidone maleate in SEDDS components.

**Fig. (3):**
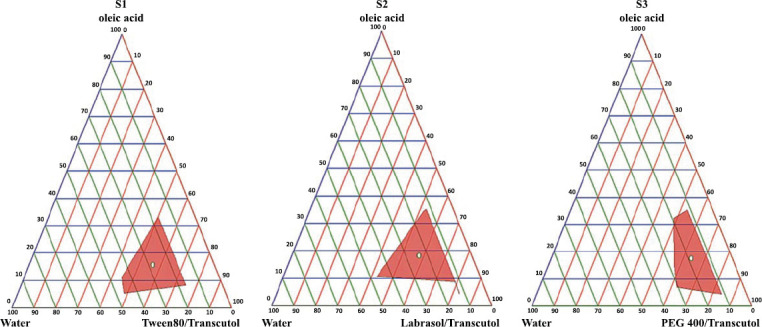
Pseudo-Ternary Phase diagrams of prepared self-emulsifying drug delivery systems. **S1**: The pseudo-ternary phase diagram of the SEDDSs composed of oleic acid, Transcutol HP, Tween 80, and distilled water. **S2**: The pseudo ternary phase diagram of the SEDDSs composed of oleic acid, Transcutol HP, Labrasol, and distilled water. **S3**: The pseudo-ternary phase diagram of the SEDDSs composed of oleic acid, Transcutol HP, PEG 400, and distilled water.

**Fig. (4):**
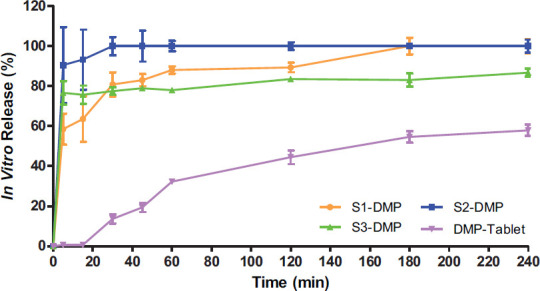
*In vitro* dissolution profiles. Formulation 1 (S1-DMP), Formulation 2 (S2 DMP), Formulation 3 (S3-DMP), Conventional Tablet (DMP-Tablet).

**Fig. (5):**
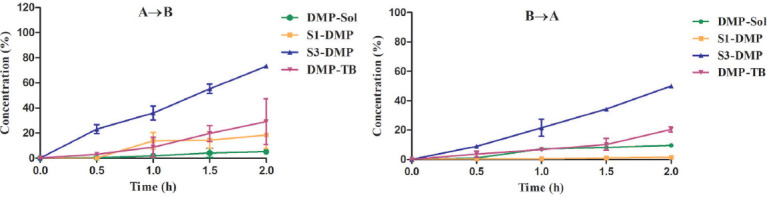
DMP concentration from SEDDSs, solutions, and commercial products permeated the monolayer of Caco-2 cells. DMP was permeated in two directions: from the apical to the basolateral side (**A** and **B**) and from the basolateral to the apical side **B** and **A**). The results are presented as the means ± SD of three experiments.

**Fig. (6):**
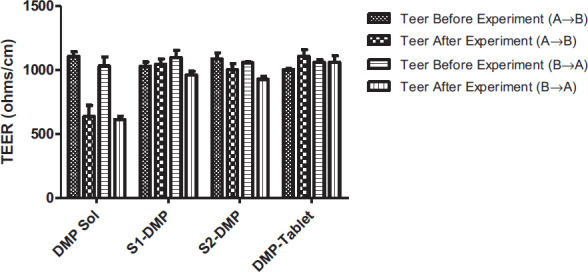
Caco-2 cell TEER (ohms/cm) change trials for apical to basolateral direction A→B, from basolateral to apical, B→A.

**Fig. (9):**
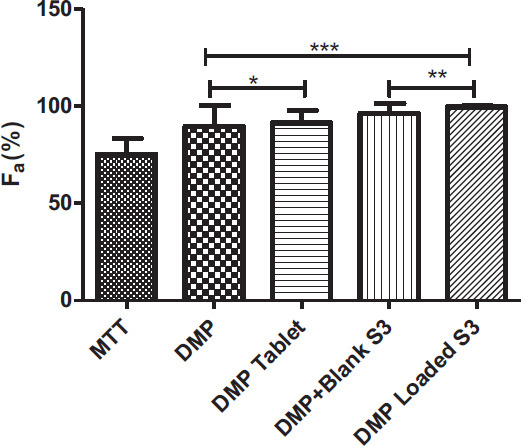
F_a_ (%) values. *p>0.05, DMP *vs.* DMP tablet; **p<0.05, DMP+Blank S3 *vs.* DMP Loaded S3; ***p<0.05, DMP *vs.* DMP+Blank S3 and DMP Loaded S3.

**Table 1:** Marker and drug concentrations in SPIP perfusion solutions.

**Drug/Marker**	**Highest Prescribed Dose (mg)**	**Concentration (mg/mL)**
PR	-^a^	0.20
MTT	100	0.40
DMP	10	0.04

**Note:** In other words, P.R. cannot be taken orally. In SPIP experiments, 0.20 mg/mL was chosen as the standard concentration of P.R. in the perfusion solution.

**Table 2:** The components of SEDDSs.

**Components (%)**	**S1**	**S2**	**S3**
**Oleic acid**	24	24	24
**Tween 80**	38	-	-
**Labrasol**	-	57	-
**PEG 400**	-	-	26
**Transcutol HP**	38	19	50
**HLB**	9.58	10.14	7.5

**Table 3:** The impact of different dispersion media and volume on formulation droplet size (nm).

**pH 1.2**					
**S1-DMP**
	**Time/Dilution Ratio**	**1:10**	**1:100**	**1:200**	**1:500**
	0. h	331.10 ± 9.20	338.00 ± 8.50	345.00 ± 7.00	367.00 ± 5.40
	4. h	345.00 ± 6.50	342.40 ± 7.30	355.00 ± 5.10	372.30 ± 6.00
	12. h	352.60 ± 9.00	365.00 ± 10.00	376.00 ± 9.70	397.00 ± 8.50
	**pH 6.8**				
	Time/dilution ratio	1:10	1:100	1:200	1:500
	0. h	320.00 ± 11.20	329.00 ± 2.80	337.00 ± 6.80	356.00 ± 11.00
	4. h	332.00 ± 5.70	341.00 ± 8.40	354.00 ± 7.40	368.00 ± 10.00
	12. h	344.00 ± 3.80	359.00 ± 7.50	361.00 ± 9.40	372.00 ± 9.80
**S2-DMP**	**pH 1.2**				
	**Time/Dilution Ratio**	**1:10**	**1:100**	**1:200**	**1:500**
	0. h	251.80 ± 12.30	255.50 ± 5.70	261.60 ± 6.40	272.60 ± 4.60
	4. h	268.80 ± 2.50	271.20 ± 9.40	278.30 ± 3.70	282.00 ± .9.60
	12. h	271.00 ± 12.00	283.00 ± 6.80	288.20 ± 8.60	290.00 ± 8.80
	**pH 6.8**				
	**Time/Dilution Ratio**	**1:10**	**1:100**	**1:200**	**1:500**
	0. h	242.10 ± 5.40	244.50 ± 9.70	257.30 ± 9.50	262.00 ± 9.90
	4. h	250.00 ± 6.80	254.30 ± 12.00	260.00 ± 4.80	267.30 ± 8.60
	12. h	256.00 ± 8.70	260.20 ± 12.30	269.30 ± 11.00	273.00 ± 4.70
**S3-DMP**	**pH 1.2**				
	**Time/Dilution Ratio**	**1:10**	**1:100**	**1:200**	**1:500**
	0. h	553.60 ± 3.60	562.60 ± 9.70	571.00 ± 9.90	578.00 ± 11.00
	4. h	561.00 ± 6.80	574.00 ± 6.80	583.00 ± 7.80	596.00 ± 13.50
	12. h	574.00 ± 10.20	579.30 ± 11.00	593.50 ± 4.70	601.00 ± 15.00
	**pH 6.8**				
	**Time/Dilution Ratio**	**1:10**	**1:100**	**1:200**	**1:500**
	0. h	482.50 ± 11.20	493.00 ± 10.00	499.00 ± 7.80	503.00 ± 9.80
	4. h	487.00 ± 10.60	500.00 ± 2.80	503.00 ± 5.70	507.80 ± 11.60
	12. h	498.00 ± 13.50	507.20 ± 4.70	512.50 ± 4.60	523.00 ± 2.50

**Table 4:** The permeability values with S.D. of commercial tablets, DMP solution, and S1-DMP and S3-DMP formulations. The permeability value for the A→B direction is Papp (A→B). Papp (B→A), permeability value for the B→A direction.

**Formulation/Papp**	**DMPSOL**	**S1-DMP**	**S3-DMP**	**DMP-Tablet**
**Papp (x10-4)** (A→B)	1.56 ± 0.93	3.31 ± 1.57	12.74 ± 0.02	5.34 ± 3.04
**Papp (x10-4)** (B→A)	9.13 ± 0.02	0.03 ± 0.02	8.93 ± 0.078	3.31 ± 0.02
**Papp** (B→A) **Papp** (A→B) Ratio	5.85	0.09	0.70	0.62

**Table 5:** TEER values and percentage of change of SEEDS formulations, DMP solution, commercial tablet with ± S.D. for A→ B direction and B→ A direction.

**TEER A→ B (ohms/cm)**			
**Formulations**	**Before**	**After**	**Change (%)**
DMP-SOL	1107.222 ± 35.766	637.100 ± 86.748	(-)42.5
S1-DMP	1029.556 ± 34.162	1045.333 ± 42.503	(+)1.5
S3-DMP	1087.889 ± 47.214	1001.667 ± 48.466	(-)7.9
DMP-Tablet	1002.778 ± 8.075	1107.867 ± 52.313	(+)10.5
**TEER B→ A (ohms/cm)**			
**Formulations**	**Before**	**After**	**Change (%)**
DMP-SOL	1032 ± 71.506	611.367 ± 28.453	(-)40.7
S1-DMP	1096.667 ± 55.935	961.667 ± 29.071	(-)12.3
S3-DMP	1058.222 ± 6.354	928.000 ± 22.145	(-)12.3
DMP-Tablet	1061.667 ± 21.064	1060.444 ± 51.896	(-)0.12

**Table 6:** The net water flux (NWF) in each group (𝑛 = 6).

**Time (min.)**	**DMP**	**DMP Tablet**	**DMP+Blank S3**	**DMP Loaded S3**
20	-41 ± 44	-30 ± 17	74 ± 17	6 ± 28
30	-73 ± 60	-32 ± 26	-24 ± 62	-42 ± 63
40	-50 ± 31	-20 ± 29	21 ± 49	-28 ± 39
50	-35 ± 55	-55 ± 82	41 ± 30	-22 ± 36
60	-34 ± 66	-39 ± 39	3 ± 46	-63 ± 56
Mean ± SD	-47 ± 35	-35 ± 13	23 ± 18	-30 ± 14

**Table 7:** Permeability of DMP (𝑛 = 6).

**Drug/System**	**P_eff_** **(*x*10^-4^, *cm/s*)**	**k_a_** **(*x*10^-4^, 1/*s*)**	**ER**
MTT	0.376 ± 0.092	13.088 ± 1.637	-
DMP	0.802 ± 0.418	15.948 ± 4.231	1.000
DMP Tablet	0.616 ± 0.354	13.822 ± 3.144	0.768
DMP +Blank S3	0.882 ± 0.471	17.352 ± 6.121	1.100
DMP Loaded S3	2.122 ± 0.892	22.539 ± 6.464	2.650

## Data Availability

Not applicable.

## LIST OF ABBREVIATIONS

ATCC: American Type Culture Collection
DMEM: Dulbecco's Modified Eagle medium
HBSS: Hank's Balanced Salt Solution
SEDDS: Self- Emulsifying Drug Delivery System
